# Clinical Holistic Medicine: The Dean Ornish Program (“Opening the Heart”) in Cardiovascular Disease

**DOI:** 10.1100/tsw.2006.330

**Published:** 2006-02-02

**Authors:** Søren Ventegodt, Efrat Merrick, Joav Merrick

**Affiliations:** ^1^ Nordic School of Holistic Medicine and Quality of Life Research Center, Teglgå rdstræde 4-8, DK-1452 Copenhagen K, Denmark; ^2^ The Scandinavian Foundation for Holistic Medicine, Sandvika, Norway; ^3^ National Institute of Child Health and Human Development, Israel; ^4^ Center for Multidisciplinary Research in Aging, Faculty of Health Sciences, Ben Gurion University of the Negev, Beer-Sheva, Israel; ^5^ Office of the Medical Director, Division for Mental Retardation, Ministry of Social Affairs, Jerusalem, Israel

**Keywords:** quality of life, QOL, philosophy, human development, holistic medicine, public health, holistic health, holistic process theory, life mission theory, group therapy, Denmark

## Abstract

Dean Ornish of the Preventive Medicine Research Institute in Sausalito, California has created an intensive holistic treatment for coronary heart patients with improved diet (low fat, whole foods, plant based), exercise, stress management, and social support that has proven to be efficient. In this paper, we analyze the rationale behind his cure in relation to contemporary holistic medical theory. In spite of a complex treatment program, the principles seem to be simple and in accordance with holistic medical theories, like the Antonovsky concept of rehabilitating the sense of coherence and the life mission theory for holistic medicine. We believe there is a need for the allocation of resources for further research into the aspects of holistic health and its methods, where positive and significant results have been proven and reproduced at several sites.

## INTRODUCTION

Holistic medicine can be an efficient tool in cases where the goal of intervention is in the improvement of global quality of life (QOL), health, and ability of the patient[1,2,3,4,5,6,7,8,9,10,11]. In the past decade, holistic medicine with a focus on improving the quality of life of the patient has shown remarkable results in several studies published in the most recognized medical journals[12,13], even with cancer and coronary hearth disease. The field has been difficult to approach from a theoretical perspective, as the depth of consciousness and experience is still out of the reach of established medical science. In spite of this, several theories of the connection between quality of life and health seem to be very meaningful and also documented to some extent, such as the work of Aaron Antonovsky (1923–1994) on sense of coherence[14,15,16,17], the work of Abraham Maslow (1908–1970) on needs[18], and Viktor Frankl (1905–1997) on meaning[19]. Our own theory of the purpose of life (the life mission theory) has tried to crystallize the essence on the basis of the work done by these pioneers[20,21,22,23,24,25,26].

Everybody will eventually die and most from coronary vessel failure. It is extremely interesting that Dean Ornish, MD, has directed clinical research for the past 25 years demonstrating that comprehensive lifestyle changes may begin to reverse even severe coronary heart disease, without drugs or surgery, and it seems that he is able to heal at least a fraction of these patients[13,27,28,29]. (Dr. Ornish is founder, president, and director of the nonprofit Preventive Medicine Research Institute in Sausalito, California, where he holds the Bucksbaum Chair and is Clinical Professor of Medicine at the University of California, San Francisco. He received his medical training from Baylor College of Medicine, Harvard Medical School, and Massachusetts General Hospital.) In this paper, we intend to analyze his cure for coronary heart disease to see if we can find it related to and substantiated by contemporary holistic medical theory, in the same way as we have analyzed the work of Ryke Geerd Hamer, MD on cancer[30].

## CLINICAL HOLISTIC MEDICINE

The life mission theory[20,21,22,23,24,25,31,32,33,34,35,36] states that everybody has a purpose of life or a huge talent. Happiness comes from living this purpose and succeeding in expressing the core talent in your life. To do this, it is important to develop as a person into what is known as the natural condition, a condition where the person knows himself and uses all his efforts to achieve what is most important for him. The holistic process theory of healing[37,38,39,40] and the related quality of life theories[41,42,43] state that the return to the natural state of being is possible whenever the person receives the resources needed for existential healing. The resources needed are "holding" in the dimensions of awareness, respect, care, acknowledgment, and acceptance with support and processing in the dimensions of feeling, understanding, and letting go of negative attitudes and beliefs. The preconditions for the holistic healing to take place are trust and the intention for the healing to take place. Existential healing is not a local healing of any tissue, but a healing of the wholeness of the person, making him much more resourceful, loving, and knowledgeable of himself, his own needs, and his wishes. In letting go of negative attitudes and beliefs, the person returns to a more responsible existential position and an improved quality of life. The philosophical change of the person healing is often a change towards preferring difficult problems and challenges instead of avoiding difficulties in life[44,45,46,47,48,49,50,51]. The person who becomes happier and more resourceful often also becomes more healthy, more talented, and more able to function[3,4,5,52,53,54].

## PREVENTIVE MEDICINE RESEARCH INSTITUTE, SAUSALITO, CALIFORNIA

Statistically, one Dane in two will die from a cardiovascular disease. Most "heart conditions" are due to a constriction of the coronary arteries from atherosclerosis. Keeping the heart and blood vessels healthy is, therefore, very important to our life expectancy and well being. Within the last decade, holistic medicine has achieved a breakthrough in the understanding and treatment of heart conditions. With this new angle on body and soul, it is possible to make even very advanced heart disease disappear when the patients work on themselves. Many scientific measurements have shown that the constricted arteries are able to expand physically again. The pioneer within this field and a great source of inspiration is Dean Ornish.

Ornish et al.[55] published a study in 1983 to evaluate the short-term effects of intervention (stress management training and dietary changes) in patients with ischemic heart disease (IHD) who received the intervention and 23 controls. After 24 days, the experimental group had a 44% mean increase in duration of exercise, a 55% mean increase in total work performed, a 20.5% mean decrease in plasma cholesterol, and a 91% mean reduction in angina episodes. It was a breakthrough for holistic medicine in 1990, when Ornish et al.[13] demonstrated that patients can make their constricted coronary arteries expand again through change of lifestyle. This was a 1-year follow-up study of 28 patients (with 20 controls) who changed their lifestyle radically. They began to eat a low-fat vegetarian diet, they stopped smoking, and they worked in a purposeful manner to lift their stress through stress management training (meditation)

and moderate exercise. Artery lesions analyzed by quantitative coronary angiography showed regression in stenosis diameter and, overall, 82% of the experimental group had an average change towards regression, even in severe coronary atherosclerosis after 1 year without use of lipid-reducing drugs. His key tools are love, intimacy, and a new life philosophy in our interpretation of his intervention. The treatment regime affects many aspects of life. As far as we can see, the regime was designed to give the patient a feeling of being more whole and healthy. It addressed the patient's perceptions and consciousness. To us, this points to the real cause of cardiovascular disease (and of any number of other disorders and ailments) — our self-created, nonoptimal perception of ourselves, our lives, and our bodies.

In 1998, these pilot studies were further expanded with 194 in the experimental and 139 in the control group[56] and it was found that the experimental group (with the training and changes listed above) was able to avoid revascularization for at least 3 years by making comprehensive lifestyle changes at substantial low cost without increase in cardiac morbidity or mortality. At this 5-year follow-up[57], 48 patients with moderate to severe coronary disease were randomized to an intensive lifestyle change group or to the usual care group and 35 completed the 5-year follow-up quantitative coronary angiography at two tertiary care university medical centers. More regression of coronary atherosclerosis occurred after 5 years than after 1 year in the experimental group, while the control group showed coronary atherosclerosis progression and more than twice as many cardiac events.

The latest study in 2003 with 440 patients[58] (mean age 58 years, 21% women) with coronary artery disease at baseline and at 3- and 12-month follow-up showed significant improvements due to the intervention program for both genders and in both the medical and psychosocial sphere. The improvement in women was similar to that in the men, which is important because women in general have higher morbidity and mortality after a heart attack, angioplasty, or bypass surgery.

## A SCIENTIFIC EXPLANATION

Atherosclerosis is not a phenomenon that can be understood in a purely mechanical manner the way we understand lime scale in a coffee maker. Blood vessels are damaged because the cells do not work the way they should, but "scamp" and build delicate and sickly vessels. According to holistic medicine, the cells are scamping because their work is disrupted. The disrupting factors seem to be our repressed feelings acting as blockages. They are identifiable in the patient's body as muscle tensions and tender "trigger points", sites in the body that become especially tender when we restrain our feelings. These trigger points are also popular "points of attack" in the treatment for the holistic body therapist by acupressure or other techniques.

Our favorite approach is direct interaction with the patient's consciousness, where gestalts are identified and integrated. We believe that acupressure and body therapy are needed less when the patient is cooperative and willing to work on himself. Conversation is the most effective tool we know. Holistic medicine works by helping the patient to feel his blockages and old life pain in his body. Then the patient is helped to understand the correlation between body and soul in order to formulate difficult feelings. Finally, the patient will acknowledge his inappropriate decisions in life that have restrained the problems in the body up to the present. As soon as we acknowledge the perceptions and viewpoints that we harbor against life, we can let go of them and change our perspective to a happy and trusting philosophy of life, where we openly and honestly feel life as it is and accept it as a gift. Patients with a heart condition need a program that focuses on improving the quality of life with a combination of life philosophical tuition, training, and supervision. Dean Ornish uses the expression "opening the heart" — physically, emotionally, and spiritually — and the clever backbone of his course of treatment (and one that we use in our research clinic in Copenhagen) is an individual program that combines holistic medical treatment with a personal development program for the patient to carry out on his or her own. The patient who has experienced heart problems before is offered "secondary prevention" in the form of a personal development program that should counteract any future heart conditions.

## CASE STORIES

> **Male, aged 43 years, with heart problems:** For a month he has had transient attacks during which he felt poorly with pain in right arm and right side of the head. He believes that his heart is not beating quite the way it should. He also has some existential aspects that need looking at. On examination: Auscultation of the heart, lungs and blood pressure: normal. Trigger points in chest, arms and legs consistent with chronic stress that can damage the heart. Prescribe exercise ECG and another appointment in three weeks. EXERCISE: Find out what you can improve in your life.

The electrocardiogram did not show much unless the patient was physically challenged, when it is called an exercise ECG. In our experience, the short exercise in how to improve life can solve stress-related heart conditions like the one in hand rapidly and effectively. Actually, we do not know whether it is the heart or the muscles causing the symptoms. In this case, the doctor could not provoke pain by pressing on the muscles. This led us to suspect that it may, in fact, be the heart causing the symptoms. Very mild cardiac disturbances are unlikely to be captured on an ECG. As an exception, below we bring a case record that is not our own, but was written by a Rosen practitioner at our instigation. It is instructive and shows important aspects of how to heal your heart.

> **Male, aged 56 years, with heart problems:** This patient is in his mid-50s, a family man and manager of a private firm. He seems a happy and extrovert man with a good grip on things. However, his body was heavy and his muscles were very hard. Shortly before he started at my clinic, he had been in hospital with a blood clot in his heart and prescribed medication for hypertension. Most of the times on the couch he fell into a deep sleep that was frequently interrupted by some very violent jerks throughout the body, which he called his electric shocks. Several times during the period, when he came to see me he was admitted with extreme cardiac pain and angina. And eventually he was given medication for these symptoms and put down for bypass surgery. During some of his private sessions he became aware of some of the things that had greatly influenced his life, including an alcoholic father, who was violent towards his mother and the fact that when he was very young he had had electroconvulsive therapy for severe depression. After he had realised this, the jerks that used to wake up both him and his wife ceased or diminished. It also became apparent to me that he was taking strong antidepressants and had done so for years. He now chose to reduce dosage so that he was far below the daily dose and he was doing well without the excessive medication. Throughout the therapy he had some major problems with his staff, which he felt had taken a dislike to him. The patient mobilised all his strength to give notice and start again from scratch in another firm, where he is working today. At some point he was again admitted with extreme pain and angina that the hospital considered to be life-threatening, so he was transferred to a cardiology ward for surgery at the earliest opportunity. However, when the cardiologists examined him thoroughly they could not find any disorder or defect in the heart or surrounding blood vessels, so they discharged him again. During my last private session with the patient he was truly happy about life and full of vigour to devote himself to his family and friends. His jerks and cardiac problems had vanished completely, and he was enjoying his new job.

The Rosen method and other body therapies that make the patient note the feelings located in the body are effective tools in holistic medicine. Sometimes the patient can verbalize his feelings and let go. For many middle-aged men, their “Achilles’ heal” is allowing themselves to feel. Often, it is extremely unpleasant for a grown-up man in a managerial position to register the old feelings from his childhood of being small, frightened, and helpless with a feeling that it is an insult to his ego that he is still harboring such emotions. To release them is good for the heart.

## DISCUSSION

Ornish et al.[13,28,29,55,56,57] have shown that when patients with heart conditions caused by severe atherosclerosis change their lives and start to meditate, the constricted blood vessels can expand again. In the controls, the blood vessels continued to constrict and soon these patients will need bypass surgery to graft new vessels in their hearts. Often after surgery, the new vessels will also narrow and block in time. A new lifestyle seems to be the only lasting solution to this problem. So far, the most troubling problem seemed to be that the success of Ornish et al. to induce these healings have not been repeated by other medical teams. It is well known that the charisma of a therapist can be so enormous that this therapist can make almost all cures work, even when treating the patient with poisonous drugs like arsenic, which was often used as medicine only a century ago. Dean Ornish is known to be a man of such charisma and it may be he, more than his treatments, that actually cures the patients.

In order to test this, it was urgently needed that multicenter studies be made using the Ornish program for "opening the heart", which actually was done with the study in 2003[58]. This study examined both medical and psychosocial aspects of 440 patients (mean age 58 years, 21% women) with coronary artery disease at baseline and a 3- and 12-month follow-up. All were part of a multicenter Lifestyle Demonstration Project, where the participants improved diet (low fat, whole foods, plant based), exercised, learned stress management, and received social support. Partners were also asked to participate in order to maximize the effect on the family unit. Both genders had significant improvements in their diet, exercise, and stress management practices. These improvements were maintained over the 12-month course of the study. Both women and men also showed significant medical (e.g., plasma lipids, blood pressure, body weight, exercise capacity) and psychosocial (e.g., quality of life) improvements. This multicenter study showed that a multicomponent lifestyle change program can be implemented successfully and repeated at various sites.

It seems that the results of the clinical work of Dean Ornish as a whole, on patients with coronary hearth disease, is fairly well explained by contemporary holistic medical theory. When a person heals emotionally and these emotions are connected to blockages in the heart region of the body, all tissues in this region might be affected. Since the coronary vessels are the weakest link, they break down first. When the person integrates the feelings giving informational disturbance to the tissues, the tissues will heal and so the coronary vessels can open again as the cells aging receive correct information on structure and functioning from the information system of the body[47].

## ACKNOWLEDGMENTS

This study was supported by grants from IMK Almene Fond. The quality of life research was approved by the Copenhagen Scientific Ethical Committee under number (KF)V.100.2123/91.

## BIOSKETCHES

**Søren Ventegodt, MD,** is the director of the Quality of Life Research Center in Copenhagen, Denmark. He is also responsible for a Research Clinic for Holistic Medicine in Copenhagen and is a popular speaker throughout Scandinavia. He has published numerous scientific or popular articles and a number of books on holistic medicine, quality of life, and quality of working life. His most important scientific contributions are the comprehensive SEQOL questionnaire, the very short QoL5 questionnaire, the integrated QOL theory, the holistic process theory, the life mission theory, and the ongoing Danish Quality of Life Research Survey, 1991–94 in cooperation with the University Hospital of Copenhagen and the late professor of pediatrics, Bengt Zachau-Christiansen, MD, PhD. E-mail: ventegodt@livskvalitet.org. Website: http://www.livskvalitet.org

**Efrat Merrick** is a medical student at the Sackler School of Medicine, Tel Aviv University and a research assistant at the National Institute of Child Health and Human Development, Faculty of Health Sciences, Ben Gurion University of the Negev, Beer-Sheva, Israel. E-mail: efratmerrick@gmail.com

**Joav Merrick, MD, DMSc,** is professor of child health and human development affiliated with the Center for Multidisciplinary Research in Aging, Zusman Child Development Center, Division of Pediatrics and Community Health at the Ben Gurion University, Beer-Sheva, Israel; the medical director of the Division for Mental Retardation, Ministry of Social Affairs, Jerusalem; and founder and director of the National Institute of Child Health and Human Development. He has numerous publications in the field of child health and human development, rehabilitation, intellectual disability, disability, health, welfare, abuse, advocacy, quality of life and prevention. Dr. Merrick received the Peter Sabroe Child Award for outstanding work on behalf of Danish Children in 1985 and the International LEGO-Prize ("The Children's Nobel Prize") for an extraordinary contribution towards improvement in child welfare and well being in 1987. E-mail: jmerrick@internet-zahav.net. Website: www.nichd-israel.com
